# Surface Nanostructures Formed by Phase Separation of Metal Salt–Polymer Nanocomposite Film for Anti-reflection and Super-hydrophobic Applications

**DOI:** 10.1186/s11671-017-2402-4

**Published:** 2017-12-16

**Authors:** Celal Con, Bo Cui

**Affiliations:** Waterloo Institute for Nanotechnology (WIN), University of Waterloo, 200 University Ave. West, Waterloo, Ontario N2L 3G1 Canada

**Keywords:** Surface structures, Self-assembly, RIE, Metal salt, Anti-reflectivity, Super-hydrophobicity

## Abstract

This paper describes a simple and low-cost fabrication method for multi-functional nanostructures with outstanding anti-reflective and super-hydrophobic properties. Our method employed phase separation of a metal salt–polymer nanocomposite film that leads to nanoisland formation after etching away the polymer matrix, and the metal salt island can then be utilized as a hard mask for dry etching the substrate or sublayer. Compared to many other methods for patterning metallic hard mask structures, such as the popular lift-off method, our approach involves only spin coating and thermal annealing, thus is more cost-efficient. Metal salts including aluminum nitrate nonahydrate (ANN) and chromium nitrate nonahydrate (CNN) can both be used, and high aspect ratio (1:30) and high-resolution (sub-50 nm) pillars etched into silicon can be achieved readily. With further control of the etching profile by adjusting the dry etching parameters, cone-like silicon structure with reflectivity in the visible region down to a remarkably low value of 2% was achieved. Lastly, by coating a hydrophobic surfactant layer, the pillar array demonstrated a super-hydrophobic property with an exceptionally high water contact angle of up to 165.7°.

## Background

In recent decades, there is an increasing demand on surface nanostructures for their effect on the properties of the underneath bulk materials. These structures are often called “smart” coatings where they could provide enhanced functionalities such as wetting/de-wetting, thermal and/or electrical conductivity, super-hydrophobicity, self-cleaning, anti-icing, anti-reflectivity, capability to direct cell growth, and gas barrier properties [1–4]. Those structures are generally periodic pillars, cones, or porous. Yet, a recent study also brought attention to random structures that offer new degrees of freedom and possibilities by the control of their statistical properties [5] .

One common application of these structures is anti-reflectivity for solar cells, light-emitting diodes, camera lenses, glass windows, etc., where the reflection of incident light from the substrate surface is greatly reduced to improve their efficiency. Super-hydrophobicity is another important function of these structures as there are numerous industrial applications based on their self-cleaning lotus leaf effect. Both anti-reflective and super-hydrophobic effects may be observed on the same surfaces having small structures, which could be used on many levels of todays’ technology. In nature, this is already observed in a moth’s eye covered by a quasi-periodic array of sub-wavelength structures that enables it to hide from its predators as well as to keep particle and liquid away from its eye and hence enhance its vision [6] *.*

To mimic the nature and fabricate structures with both anti-reflective and hydrophobic properties, top-down nanopatterning techniques including optical lithography [7], electron beam lithography [8], and nanoimprint lithography [9] have been utilized [10–12]. However, they are costly processes. On the other hand, bottom-up techniques that are commonly called self-assembly have much lower cost than top-down techniques, though it can only achieve either random or periodic patterns without long-range ordering. Nanosphere lithography is one popular bottom-up fabrication technique where nanosized spheres are assembled to form periodic structures, yet it is challenging to form a uniform monolayer sphere for sub-100 nm sphere size [13]. Di-block copolymer lithography is another popular bottom-up technique, yet it can be lengthy and very sensitive to substrate preparation, and a feature size over 100 nm is hard to obtain. In recent years, self-masking effect in reactive ion etching that leads to black silicon via texturing the surface has been reported [14–18]. Such texturing or roughness occurs due to deposition caused by sputtering of materials from the metallic or dielectric reactor wall, which then acts as hard micro-etching mask during substrate etching. Yet, this technique generally requires a specific etching system or complex process that limits its choice of substrate materials [17, 19]. Another popular technique is the deposition or coating of a metallic film on a substrate followed by thermal annealing to achieve sub-micrometer-sized metal islands that can be used as hard mask for etching the substrate [20–25]. Yet, such island film formation needs high cost vacuum deposition and/or high annealing temperature conditions that limits their usage.

Previously, we have shown a simple process using low-cost spin-coating method and reactive ion etching pattern transfer technique to obtain surface nanostructures [26]. In this work, we extended the choice of metal salts and optimized the process, to achieve sub-20-nm resolution nanostructures over a large area. Compared to other fabrication methods, ours is a promising technique for fabricating surface nanostructures with very low-cost and high resolution. In addition, we demonstrated remarkable anti-reflective and hydrophobic properties of such structures.

## Methods/Experimental

We have investigated several metal salts that can be used as a hard mask for dry etching. Nickel salt has been previously studied [26]. However, nickel is a magnetic material and not allowed in dry etchers in many cleanrooms. Here, we extended the choice of metal salts that is more compatible with high dry etching selectivity. Aluminum and chromium are the two most commonly used metal hard mask materials for pattern transferring, so their salts were chosen in this study. For instance, selectivity between Cr and Si using non-switching pseudo-Bosch process that gives very smooth and vertical sidewall can reach 1:100 [27]. These metals are found in the form of metal salts such as aluminum (III) nitrate nonahydrate [Al(NO_3_)_3_·9H_2_O] (ANN) and chromium (III) nitrate nonahydrate [Cr(NO_3_)_3_·9H_2_O] (CNN). ANN and CNN have low melting points of 66 and 60 °C, respectively, which increase the chance of phase separation of the salt–polymer mixture at relatively low temperature. Additionally, it is found that, similar to nickel metal salts, those metal salts are soluble in dimethylformamide (DMF) solvent which we have used in our previous work. Hence, both ANN and CNN are investigated here.

In the experiment, we first dissolved poly(methyl methacrylate) (PMMA) powder (996 kg/mol, Sigma Aldrich) with 10 wt./vol.% concentration in DMF. In parallel, we dissolved ANN or CNN (99.999% purity, Sigma Aldrich) in DMF with varying concentrations of 1–10 wt./vol.%. Afterwards, we mixed the as-prepared PMMA solution and the salt solution with 1:1 volume ratio and obtained a uniform clear solution. As such, the final solution for spin-coating contains 0.5–5 wt./vol.% metal salt and 5 wt./vol.% PMMA, leading to a weight/volume ratio of metal salt and PMMA ranging from 1:10 to 10:10. The solution in DMF was homogeneous to give a smooth thin film after spin-coating on a substrate. The reason for choosing DMF as the solvent can be found in our previous work [26]. It is known that metal salts are generally soluble in water whereas polymers are soluble in organic solvents such as benzene, toluene, and tetrahydrofuran (THF). We studied several solvents and found out that our metal salts are soluble in THF, acetic acid, and DMF, which also dissolve PMMA powder. We finally chose DMF solvent because it gives more uniform and smooth salt-PMMA composite film upon spin-coating and thermal annealing process.

**Fig. 1:**
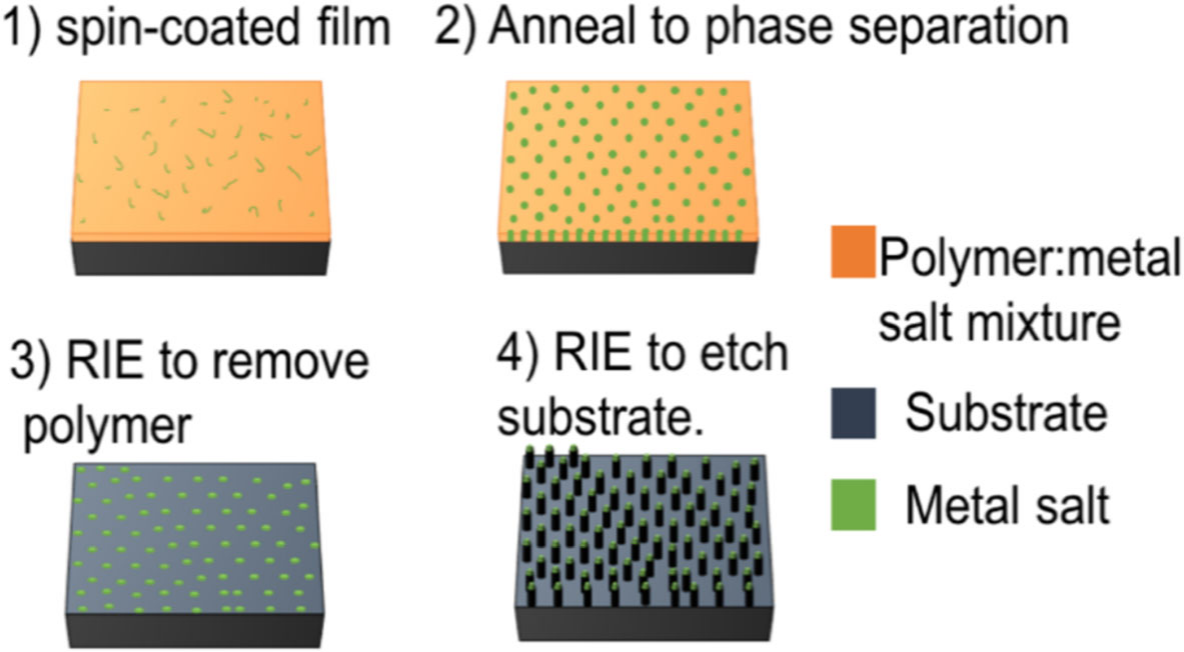
Fabrication process of ultra-high resolution nanostructures using self-assembly of metal salt–polymer nanocomposite film. (1) Spin-coating film from a solution containing polymer and salt. (2) Phase separation by thermal annealing. (3) Etching polymer using oxygen plasma and leaving behind metal salt nanoislands on silicon. (4) Etching silicon using fluorine based plasma with metal salt nanoislands as mask

**Fig. 2:**
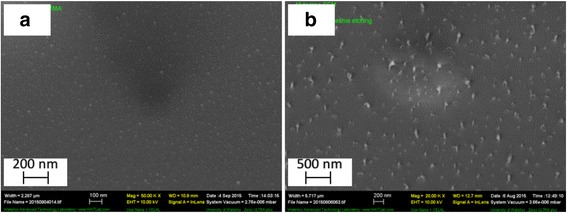
SEM image leftovers of PMMA film on silicon substrate upon oxygen plasma treatment. **a** Result of oxygen plasma process on pure PMMA film and **b** metal salt-containing PMMA film after 10-min oxygen plasma etching

The fabrication process for nanostructured silicon as an example is shown in Fig. 1. We cleaned the silicon substrate by solvent and oxygen plasma and coated 100 nm PMMA on silicon. This layer of pure PMMA film was found to help attain a more uniform film of the PMMA-salt nanocomposite film. Then, the mixture was spin-coated on PMMA film, to obtain a 300-nm-thick film for the case with 10:1 weight ratio of PMMA:metal (obtained by mixing at equal volume 10 wt./vol.% PMMA solution and 1 wt./vol.% salt solution, both in DMF). Previously, it was discussed that there is a negligible intermixing between polymer–metal salt composite and the bottom PMMA layer during the spin-coating process [26]. Next, thermal annealing was carried out to induce phase separation between polymer and metal salt. As a last step, dry etching was carried out to first etch away the polymer matrix using oxygen plasma, leaving behind metal salt nanoislands on silicon as seen in Fig. 2, then into the silicon substrate using SF_6_/C_4_F_8_ plasma. Here, the silicon pillars are formed by dry etching with metal salt nanoislands as mask, which is very different from the black silicon [14]. Those structures are formed due to micro-masking effect with the micro-mask formed in-site during the plasma etching process. Indeed, no pillars were formed using pure PMMA (no metal salt) as mask, further confirming the metal absence of micro-masking effect in our process.

## Results and Discussions

### Effect of Annealing Temperature on the Formation of Nanoislands

**Fig. 3:**
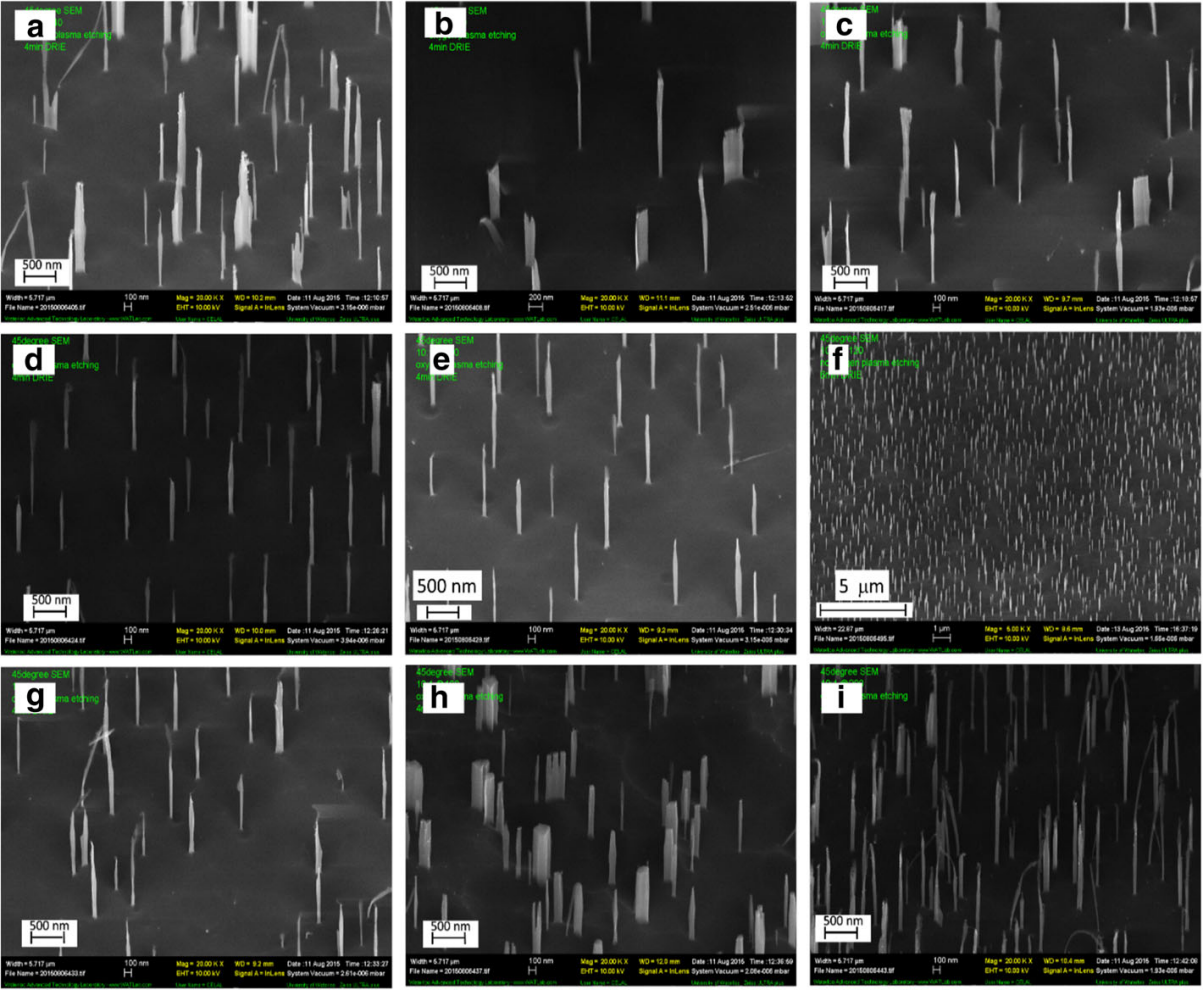
SEM images of silicon nanostructures with 1:10 ANN:PMMA ratio annealed at different temperatures. **a** 40 °C, **b** 50 °C, **c** 80 °C, **d** 100 °C, **e** 120 °C, **f** 120 °C, low magnification, **g** 150 °C, **h** 180 °C, and **i** 200 °C

To study the effect of annealing temperature on phase separation of the nanocomposite film, we prepared ANN:PMMA with 1:10 ratio. Keeping the same spin-coating conditions, we annealed the films at temperatures ranging from 40 to 200 °C for 1 h. After annealing, the samples were exposed to oxygen plasma to remove the polymer matrix from the film, and then, the underneath silicon was etched using a non-switching etching recipe with SF_6_ and C_4_F_8_ gas. Typical resulted structures are shown in Fig. 3. Nanopillars were formed in all conditions, and a relatively uniform distribution of pillar diameter and inter-pillar spacing was obtained when the film was annealed at 120 °C (Fig. 3e, f).

### Effect of Metal Salt: Polymer Ratio on the Formation of Nanostructures

**Fig. 4:**
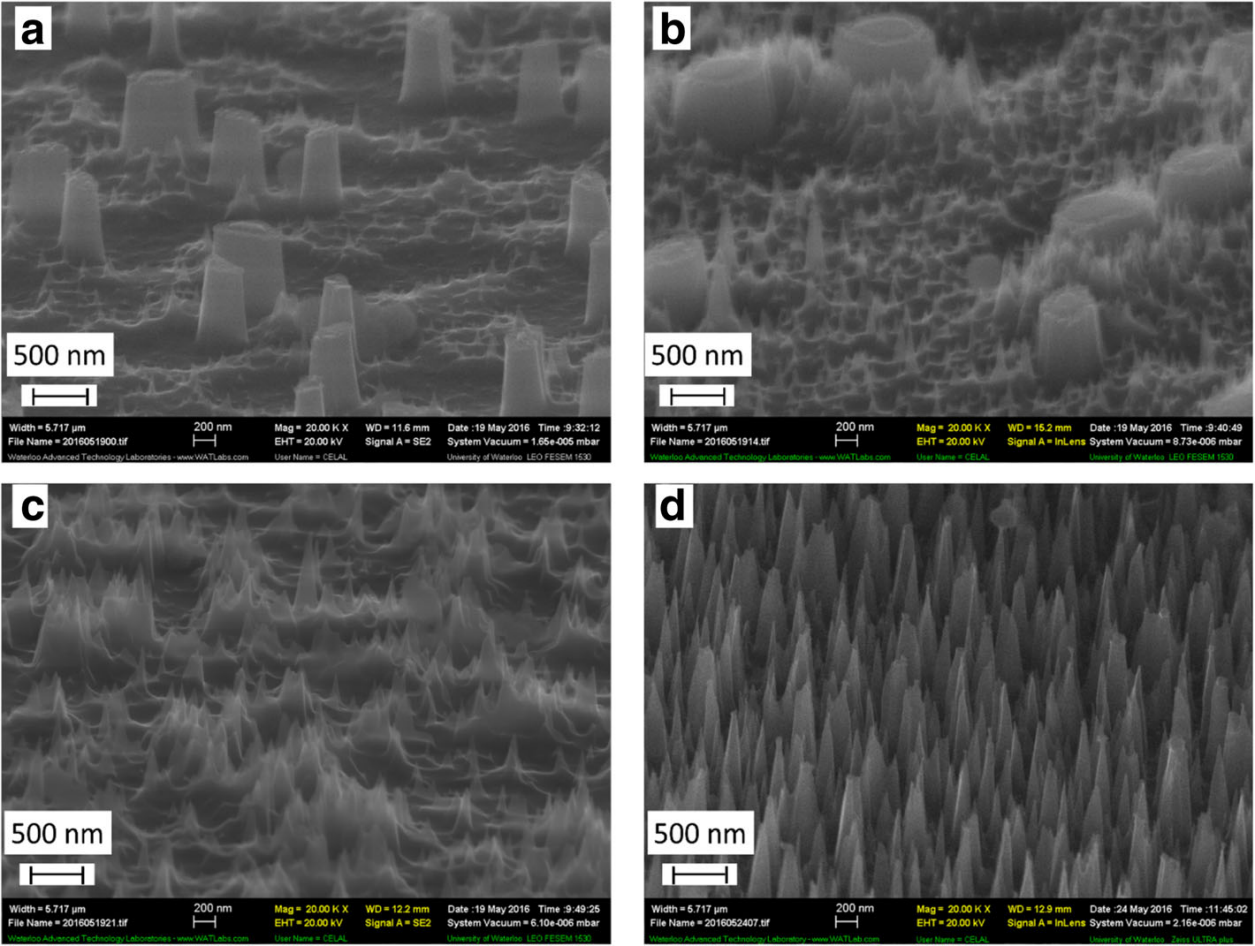
SEM images of silicon nanopillars formed upon RIE using our process with aluminum nitrate nonahydrate: polymer. Ratio of aluminum metal salt: PMMA is **a** 1:10, **b** 2:10, **c** 3:10, and **d** 5:10

**Fig. 5:**
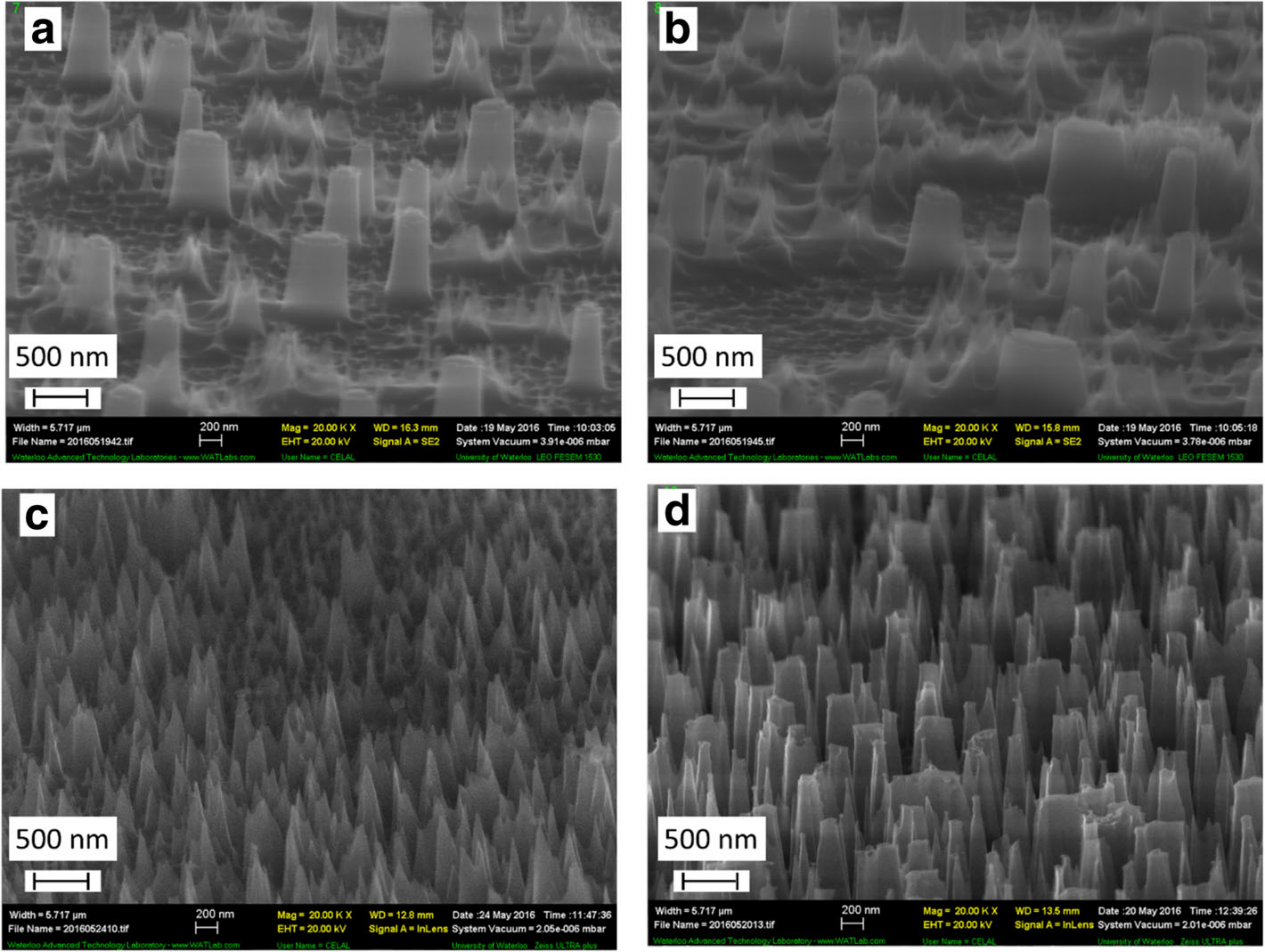
SEM images of silicon nanopillars formed upon RIE using our process with chromium nitrate nonahydrate: polymer. Ratio of chromium metal salt: PMMA is **a** 1:10, **b** 2:10, **c** 3:10, and **d** 5:10

In order to apply those structures for anti-reflective or super-hydrophobic coatings, denser pillars are desired. To this end, ANN:PMMA and CNN:PMMA mixture with different ratios in DMF solvent were prepared. Upon film spin-coating on the substrate, films were baked at 120 °C for 1 . For anti-reflective and/or hydrophobic coating applications, the pillars should ideally have a cone-shape tapered sidewall profile. Hence, we modified the etching process to fabricate such cone-shaped pillars. Previously, we have reported inductively coupled plasma reactive ion etching (ICP-RIE) of silicon to give a broadly tunable tapered profile or even a negatively tapered profile (inverse cone shape) [28, 29]. Using the reported etching recipe, resulted structures are shown in Fig. 4 for ANN:PMMA and Fig. 5 for CNN:PMMA with different ratios. For ANN salt, pillars were sparse and large when the salt concentration was low and became very dense with a 100 nm diameter and a cone shape when the metal salt:polymer ratio was increased to 5:10, which would be ideal for anti-reflective applications. As for CNN salt, the pillars or cones have largely similar dimensions to those produced by ANN salt, which was expected as these two metal salts have close chemical structure and melting temperature.

**Fig. 6:**
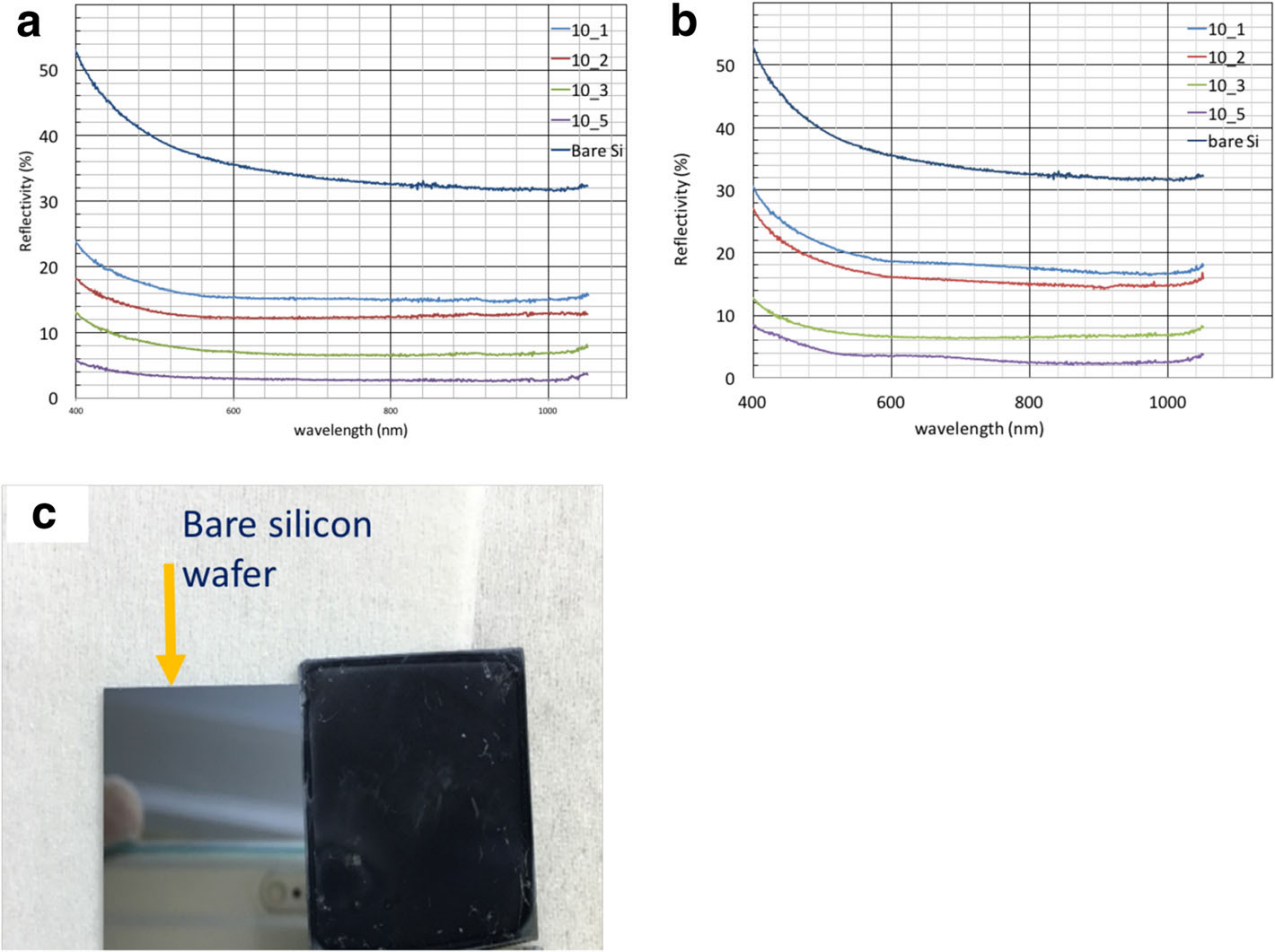
Effects of surface nanostructures formed by metal salt:PMMA film on reflectivity in visible region. **a** Reflectivity measurement of silicon wafer having nanostructures formed by using ANN:PMMA film with different metal salt–polymer weight ratios and bare silicon wafer. **b** Reflectivity measurement of silicon wafer having nanostructures formed by using CNN:PMMA film with different metal salt–polymer weight ratios and bare silicon wafer. Reflectivity is reduced to 2% by using 5:10 ratio. **c** Photo of silicon wafers before and after surface nanostructuring. Reflectivity with respect to bare silicon wafer was drastically reduced

In order to quantify the anti-reflective property, reflectivity measurements were carried out using a spectrometer (PerkinElmer Precisely Inc. Lambda 35 UV/VIS) with a spectrum scan speed of 240 nm/min. Resulted spectra are shown in Fig. 6a, b. As expected from SEM images shown in Figs. 4 and 5, reflectivity is decreased by increasing salt concentration in the nanocomposite film. Compared to bare silicon which showed ~ 35% reflectivity in the visible region, the reflectivity dropped to 15% for structures fabricated using metal salt:polymer ratio of 1:10, 12% for 2:10, 7% for 3:10, and only 2% for 5:10 ratio, which represents one order improvement over the unpatterned bare silicon wafer. Figure 6c compares silicon wafer before and after surface nanostructuring using metal salt:polymer phase separation self-assembly and RIE pattern transferring, which shows clearly the greatly reduced reflectivity for the structured surfaces.

The anti-reflectivity is rather high compared to many published results that occasionally reported reflectivity down to 2% in the visible region. Further improvement may be expected by increasing the metal salt content in the nanocomposite film, yet actually, the surface structures turned out to be very large at high metal salt content, which led to higher reflectivity. This is not surprising because more metal salt would eventually result in merged nanoislands to form much larger ones. Further enhancement of the anti-reflective property could be attained by using different plasma etching conditions to have more tapered profile or higher aspect ratio structures.

**Fig. 7:**
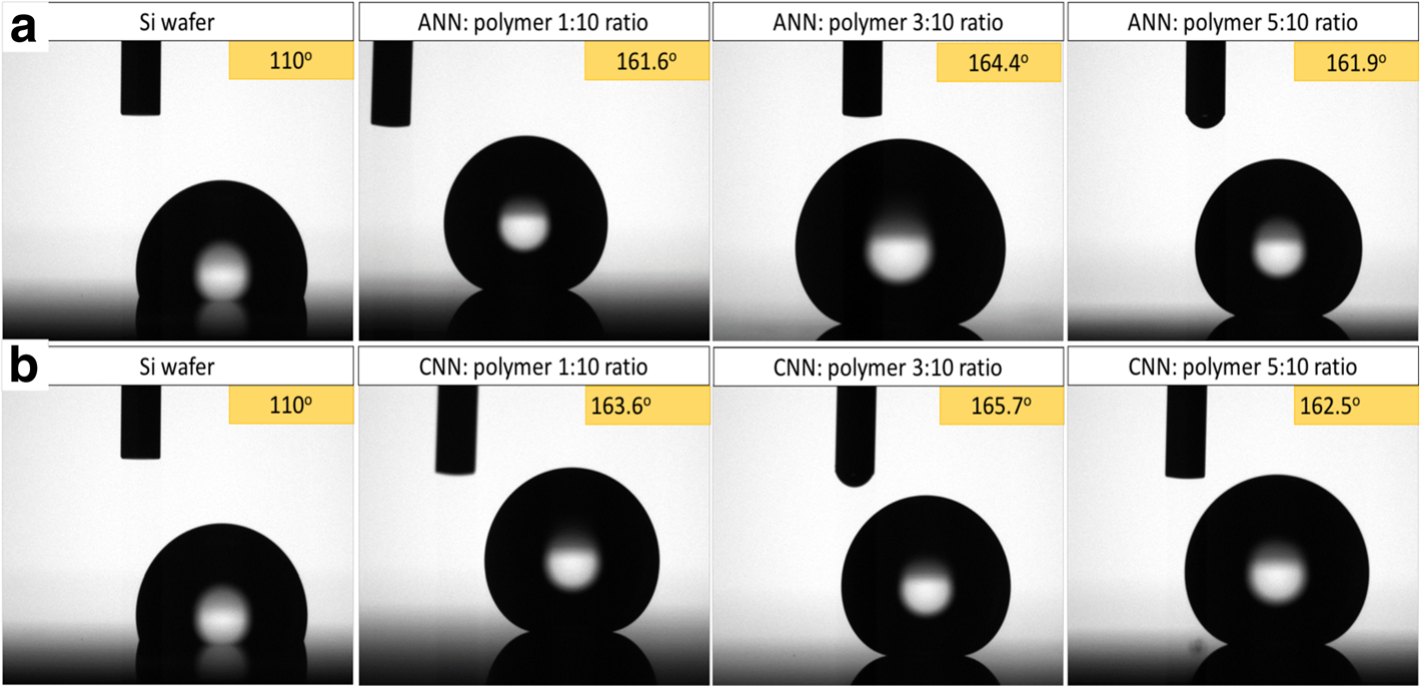
Comparison of water contact angle measurements of silicon wafer with and without nanopillars formed by metal salt–polymer film. The nanopillars were fabricated by using **a** ANN:polymer. **b** CNN:polymer metal salt with different weight ratios show super-hydrophobic properties with above 160° contact angle with the surface

Another popular application of these surface structures is for hydrophobic coatings. To study this property, water contact angle measurements were conducted using a goniometer (Ramé-hart Model 200) on samples coated with a hydrophobic self-assembled monolayer of Trichloro (1H,1H,2H,2H-perfluorooctyl) silane (FOTS) [30]. Results of water droplets on bare silicon wafer and on surface-structured wafers using metal salt (ANN or CNN):PMMA nanocomposite of different weight ratios are shown in Fig. 7. Flat silicon wafer gave a contact angle of 110° when coated with FOTS, whereas our structures can greatly increase the contact angle to a remarkable value of 165.7° when using 3:10 ratio. Our results are close to the highest reported contact angles, such as the 165° water contact angle achieved by Checco et al. [31], yet our fabrication process is simpler with lower cost.

## Conclusions

By making use of phase separation of metal salt–polymer nanocomposite film, we showed the fabrication of surface structures etched into silicon with high aspect ratio (1:30) and high resolution (sub-50 nm). Out process of patterning hard mask for further pattern transferring into the substrate has much lower cost than other traditional methods such as the liftoff process that involves metal evaporation. Aluminum nitrate and chromium nitrate can both be employed in order to obtain these structures. By using appropriate metal salt:PMMA ratio, here 5:10 as optimum, reflectivity can be drastically reduced down to only 2% for the fabricated silicon nanocone structures, which is quite remarkable for many applications. The fabricated structures can also provide super-hydrophobic property with exceptionally high water contact angle of up to 165.7°. These values could be further improved by modifying metal content in the nanocomposite film or optimizing the silicon dry etching conditions. Our results indicate that the low-cost fabrication technique is promising for applications where anti-reflection and/or hydrophobicity are critical.
